# Epidemiological and molecular characterization of a novel adenovirus of squirrel monkeys after fatal infection during immunosuppression

**DOI:** 10.1099/mgen.0.000395

**Published:** 2020-07-02

**Authors:** Donna L. Rogers, Julio C. Ruiz, Wallace B. Baze, Gloria B. McClure, Carolyn Smith, Ricky Urbanowski, Theresa Boston, Joe H. Simmons, Lawrence Williams, Christian R. Abee, John A. Vanchiere

**Affiliations:** ^1^​ Department of Pediatrics, Louisiana State University Health Sciences Center, Shreveport, LA, USA; ^2^​ Keeling Center for Comparative Medicine Research, Department of Comparative Medicine, The University of Texas MD Anderson Cancer Center, Bastrop, TX, USA

**Keywords:** adenovirus, squirrel monkey, full genome sequence, immunosuppression, fatal infection

## Abstract

Adenoviruses are a frequent cause of acute upper respiratory tract infections that can also cause disseminated disease in immunosuppressed patients. We identified a novel adenovirus, squirrel monkey adenovirus 1 (SqMAdV-1), as the cause of fatal infection in an immunocompromised squirrel monkey (*Saimiri boliviensis*) at the Keeling Center for Comparative Medicine and Research (KCCMR). Sequencing of SqMAdV-1 revealed that it is most closely related (80.4 % pairwise nucleotide identity) to the titi monkey (*Plecturocebus cupreus*) adenovirus (TMAdV). Although identified in the titi monkey, TMAdV is highly lethal in these monkeys, and they are not thought to be the natural host. While SqMAdV-1 is similar to other primate adenoviruses in size and genomic characteristics, a nucleotide polymorphism at the expected stop codon of the DNA polymerase gene results in a 126 amino acid extension at the carboxy terminus, a feature not previously observed among other primate adenoviruses. PCR testing and partial sequencing of 95 archived faecal samples from other squirrel monkeys (*Saimiri boliviensis* and *Saimiri sciureus*) housed at the KCCMR revealed the presence of three distinct, and apparently endemic species of adenoviruses. A grouping of ten squirrel monkey adenovirus variants has high similarity to SqMAdV-1. A single adenovirus variant (designated SqMAdV-3), detected in five monkeys, has similarity to tufted capuchin (*Sapajus apella*) adenoviruses. The largest group of adenovirus variants detected (designated SqMAdV-2.0–2.16) has very high similarity (93–99 %) to the TMAdV, suggesting that squirrel monkeys may be the natural host of the TMAdV.

## Data Summary

All novel sequences identified in this study have been submitted to GenBank. The accession number for the complete genome sequence of SqMAdV-1 is: MN017133. Accession numbers for the partial sequences of the novel adenoviruses identified in this study are: MN660090–MN660103 (IVa2-DNA polymerase), MN660104–MN660118 (IVa2) and MN660119–MN660140 (Hexon). The accession number for the partial IVa2-DNA polymerase sequence of common squirrel monkey adenovirus-1 is MN695333. All supporting data, code and protocols have been provided within the article or through supplementary data files. Five supplementary figures are available with the online version of this article.

**Impact Statement**

For nearly 50 years adenoviruses have been known to infect New World monkeys (NWMs; Platyrrhines), yet little is known about either the characteristics or the epidemiology of these endemic viruses. *Platyrrhini Mastadenovirus A*, isolated from a titi monkey, was the only fully sequenced adenovirus isolated from an NWM prior to this study. The fact that titi monkeys are not thought to be the natural hosts of this adenovirus highlights the scarcity of available information on endemic NWM adenoviruses. Our discovery of three distinct endemic adenovirus species circulating simultaneously in a laboratory colony of squirrel monkeys provides new insight into the diverse epidemiological characteristics of primate adenoviruses. The similarity of each of these individual species to other NWM adenoviruses expands the available data for evaluating the evolution of adenoviruses within the family Cebidae and beyond. Full genome sequencing of one of these adenoviruses, SqMAdV-1, led to the discovery of a unique feature within its DNA polymerase gene, inviting investigation into its function. These discoveries also emphasize the importance of monitoring adenoviruses in laboratory animals due to the potential for fatal infection during immunosuppression and for cross-species transmission.

## Introduction

Adenoviruses are double-stranded, non-enveloped, linear DNA viruses that infect a broad range of vertebrates, including many primates. Within the family *Adenoviridae*, there are currently more than 150 primate adenoviruses which are grouped into 17 distinct primate adenovirus species recognized by the International Committee on Taxonomy of Viruses (ICTV), and all are included within the genus *Mastadenoviruses*. Seven primate adenovirus species are designated as *Human mastadenoviruses*, nine are Old World monkey (OWM) adenovirus species, designated as *Simian mastadenoviruses,* and just one is a New World monkey (NWM) adenovirus species, designated *Platyrrhini mastadenovirus A* [1]. Mammalian adenoviruses generally have a narrow host range and are thought to have co-evolved with their primary hosts, although there are documented cases of zoonotic transmission [2, 3]. Furthermore, the high degree of phylogenetic similarity between adenoviruses of great apes and humans within the *Human mastadenoviruses* groups supports the likelihood of intraspecies recombination between adenoviruses [4, 5].

**Fig. 1.**
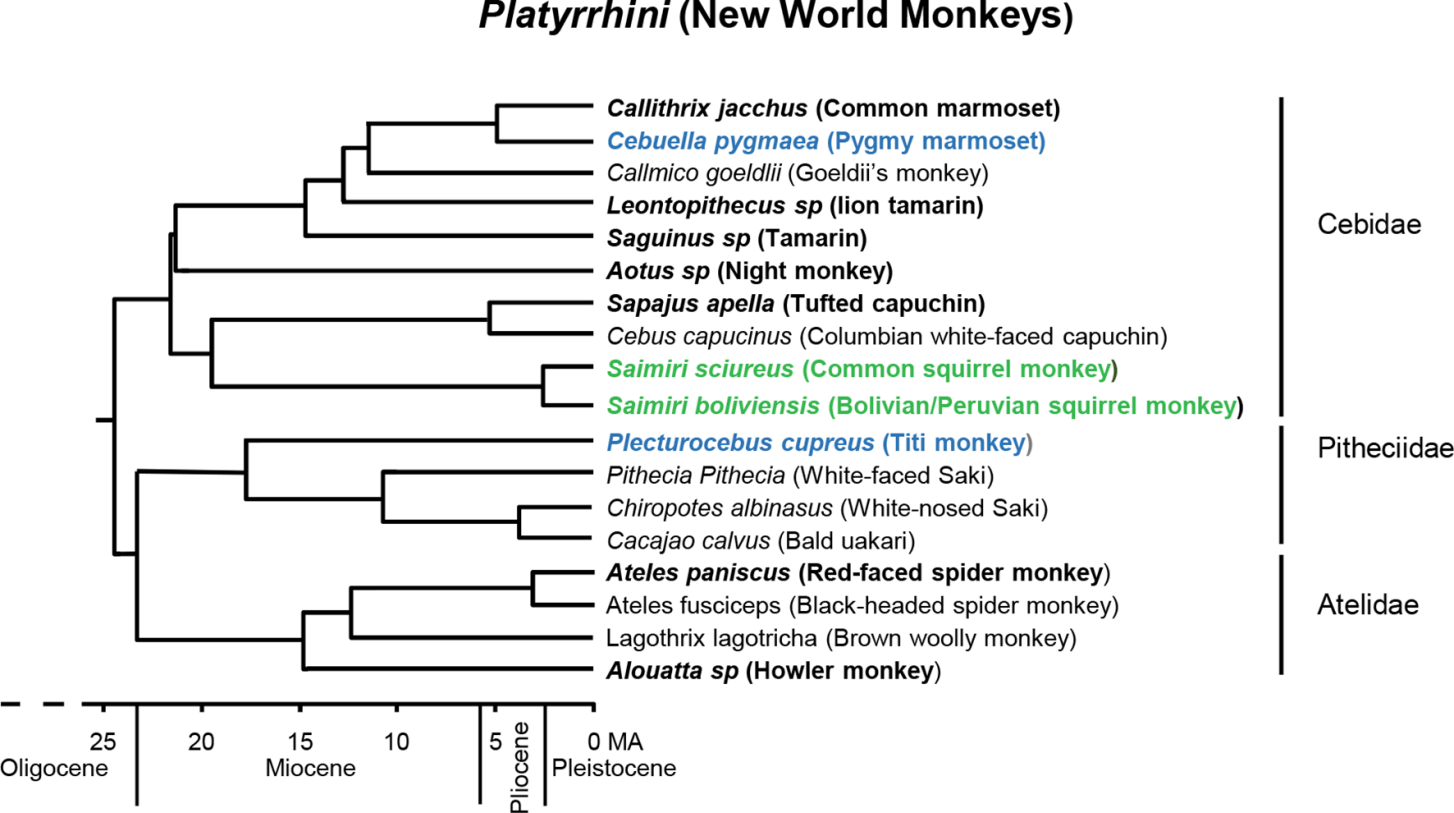
Phylogenetic tree of selected NWMs. Species in which adenovirus sequences have been reported are in bold type. Species that are not thought to be the natural host of the adenovirus detected are in bold blue type. Species in which endemic adenovirus were identified in this report are in green bold type. Adapted from *Current Zoology*, Vol. 65, Issue 5, October 2019 [41]. MA, millions of years ago.

Serological tests in common squirrel monkeys (*Saimiri sciureus*) and owl monkeys (*Aotus* sp.) reported in the 1970s provided the first evidence of adenovirus infections in NWMs [6, 7]. Two strains of adenovirus were identified in common squirrel monkeys with one strain noted to be highly infectious [6]. However, accumulated knowledge of adenoviruses in NWMs remains scant. In 2011, PCR-based detection of adenoviruses in NWMs was initially reported in captive animals, first in a research laboratory colony of titi monkeys (*Plecturocebus cupreus,* previously classified as *Callicebus cupreus*) [8], then in the common marmoset (*Callithrix jacchus*) and the white-lipped tamarin (*Saquinus labiatus),* both captive in zoological facilities [3, 5]. Collectively, adenovirus DNA detection has been reported in 14 different NWM species encompassing 10 genera: *Alouatta*, *Aotus*, *Ateles*, *Callithrix*, *Cebuella*, *Leontopithicus*, *Plecturocebus*, *Saguinus*, *Sapajus* and *Saimiri* (Fig. 1) [3, 5, 7, 9–11]. However, characterization of the adenovirus genomes identified in NWMs has been extremely limited; until now, only the titi monkey adenovirus (TMAdV) (*Platyrrhini mastadenovirus A*) has been fully sequenced, and the natural host for this adenovirus is not thought to be the titi monkey. In fact, there have been no reports of endemic adenoviruses in the Pitheciidae family of NWMs to date. In contrast, the large majority of endemic adenoviruses identified in NWMs has been reported within the family Cebidae.

Beyond the TMAdV genome, published data for NWM adenovirus DNA consist of short sequences within the DNA polymerase, hexon and IVa2 genes. With the exception of an adenovirus detected in a zoo-kept pygmy marmoset (*Cebuella pygmaea*), phylogenetic analysis conducted with the available DNA sequences convincingly separates the NWM adenoviruses from other *Mastadenoviruses*. The adenovirus identified in the pygmy marmoset (following symptoms of respiratory illness and subsequent death), based on the partial DNA polymerase gene sequence, was found to be most closely related to bat and type 2 canine adenoviruses, suggesting a cross-species transmission from an unknown reservoir [9].

In humans, adenovirus infections are common, particularly in the paediatric population [12, 13]. Adenoviruses are associated with respiratory, gastrointestinal, ocular and sexually transmitted urethral infections, and in immunocompetent individuals are usually self-limited [12, 14, 15]. In immunocompromised hosts, especially those with impaired T-cell immunity, persistent adenovirus infection can lead to a variety of clinical manifestations including pneumonia, hepatitis, haemorrhagic cystitis, colitis, pancreatitis, meningoencephalitis and disseminated disease, which can be fatal [12, 13].

Immunomodulatory therapies are an increasingly important component in the treatment of cancers, particularly haematological malignancies, and have long been essential for the success of organ transplants and for the treatment of autoimmune disorders [16–19]. Rituximab, a monoclonal antibody directed against the CD20 antigen, expressed on B cells, is a commonly used immunomodulatory therapy. Approved by the US FDA in 1997 for the treatment of non-Hodgkin’s lymphoma and in 2006 for the treatment of rheumatoid arthritis (RA), rituximab is a mainstay in the treatment of B-cell malignancies, and an important secondary line of treatment for RA patients [19–21]. Rituximab is also used off-label as an immunomodulatory therapy for renal transplant and other autoimmune disorders [22–24]. After more than 20 years of use, rituximab is considered a safe and effective immunomodulatory therapy; nevertheless, adverse events have been documented, most notably an increased risk of progressive multifocal leukoencephalopathy (PML) particularly for patients with haematological malignancies [25–27]. In contrast, reports of adenovirus disease in patients treated with rituximab are uncommon. Fatal hepatitis due to adenovirus infection in patients on rituximab therapy has been reported, one for the treatment of chronic lymphocytic leukaemia and the second for the treatment of Waldenstrom macroglobulinaemia [28, 29].

In non-human primates, adenovirus infections are typically asymptomatic; however, clinical disease in young animals, and severe disease in immunosuppressed animals has been reported [30]. Adenovirus infections have been associated with fatal pneumonia outbreaks in colonies of titi monkeys and baboons at the California National Primate Research Center (CNPRC) [2, 3]. In 1997, within the baboon colony, infants with immature immune systems were affected, and two of four infant baboons that developed respiratory infections attributed to the baboon adenovirus died [2]. In contrast, the 2009 TMAdV pneumonia outbreak occurred in healthy adult titi monkeys, devastating a colony of 65 animals [3]. During a 3 month period, 23 titi monkeys became infected, and 19 either died or were humanely euthanized. At the same time, TMAdV was not detectable in either convalescent or asymptomatic animals [3]. These circumstances strongly suggested that the titi monkey is not the natural host for the TMAdV. Testing within the CNPRC of rhesus macaque, human and rodent samples for TMAdV sequences did not identify a likely reservoir [3]. Subsequent experiments showed that the TMAdV can infect common marmosets, causing mild self-limited respiratory illness [31]. The TMAdV was also found to have infected a researcher in close contact with the sick titi monkeys and a family member of the affected researcher developed mild respiratory symptoms and was subsequently found to be seropositive for TMAdV, highlighting the risk of zoonotic and human-to-human transmission [3].

Here we describe the discovery of a novel mastadenovirus (squirrel monkey adenovirus 1, SqMAdV-1) isolated from a young squirrel monkey receiving rituximab, and characterize the SqMAdV-1 genome sequence. Additionally, we report the discovery and partial sequencing of additional adenoviruses in squirrel monkeys. Partial sequencing shows that several of these novel squirrel monkey adenoviruses are nearly identical to TMAdV, suggesting that squirrel monkeys are the natural hosts of the TMAdV.

## Methods

### Animals

All animals were housed in an Animal Biosafety Level-2-qualified research room at the Keeling Center for Comparative Medicine and Research (KCCMR), which is accredited by the Association for the Assessment and Accreditation of Laboratory Animal Care (AAALAC). All animals were born in captivity at the NIH-funded Squirrel Monkey Breeding and Research Resource within the KCCMR [32]. Ten (five male, five female) Bolivian squirrel monkeys (*Saimiri boliviensis boliviensis*), aged 3–4 months were entered into a pilot research study of immunosuppression with rituximab (Rituxan; Genentech), and inoculation with JC virus (JCV), the human polyomavirus that is the cause of PML. The course of the study included three infusions of rituximab spaced 5–9 weeks apart with the JCV inoculations occurring either 4 weeks after (Group 1) or 4 weeks before the first rituximab infusion (Table 1). The animals in each treatment group were caged together.

**Table 1.** JCV inoculation/rituximab infusion/onset of illness timeline

Group	Monkey	Week 0	Week 4	Week 9	Week 17	Week 20	Week 23
Group 1 Treatment →		Rituximab	JCV	Rituximab	Rituximab	None	None
1	SBB17						
	SBB18						
	SBB19						Ill/euthanized
	SBB20						Ill/euthanized
Group 2 Treatment→		JCV	Rituximab	Rituximab	Rituximab	None	None
2	SBB16						
	SBB21						
	SBB22				Ill/euthanized		
	SBB23					Ill/died	
Group 3 Treatment →		None	None	None	None	None	None
3	SBB14						
	SBB15						

### Post-mortem analyses

Necropsies and post-mortem analyses were performed by board-certified veterinarians specializing in non-human primate/laboratory animal medicine. At necropsy, tissues (cerebrum, cerebellum, medulla/pons, caecum, colon, duodenum, ileum, jejunum, cerebrospinal fluid, kidneys, liver, lungs, mandibular and mesenteric lymph nodes, spleen, stomach and urinary bladder) were flash frozen in dry ice. Formalin-fixed, paraffin-embedded tissues were stained with haematoxylin and eosin and evaluated by light microscopy.

### Initial virological assessments

Virological assessments of fresh-frozen tissues were performed by PCR. Using primers and thermocycling conditions developed in our laboratory (Table S1, available in the online version of this article), we tested for the JCV and for known endemic squirrel monkey viruses [33] that we had previously detected in this closed colony, including: squirrel monkey polyomavirus (SqMPyV), and three Saimiri herpesviruses, SaHV-1 (α-herpesvirus), SaHV-2 (γ-1 herpesvirus) and SaHV-3 (γ-2 herpesvirus). We tested for the presence of adenoviral DNA sequences with primers (Adeno31, Adeno32) targeting a short (161 bp) and highly conserved region of the human mastadenovirus hexon gene [34].

### Hexon gene sequencing

Primers originally developed for molecular typing of human adenoviruses (AdHexF1, AdHexR1, AdHexF2 and AdHexR2) [35, 36] were used to amplify and sequence a 757 bp section of the hexon gene near its 5′ end. From this sequence, SqMAdV-1-specific primers were developed. Similarly, the sequence of the 161 bp amplicon produced by primers Adeno31 and Adeno32 was used to develop SqMAdV-1-specific primers near the 3′ end of the hexon gene. These SqMAdV-1-specific primers were used to amplify, clone and sequence a 2 218 bp section of the hexon gene.

### Virus cultivation

Cultivation of SqMAdV-1 was conducted in A549 cells (human lung adenocarcinoma), B95a cells (an adherent derivative of the B95-8 cotton-top marmoset lymphoblast line, transformed with Epstein–Barr virus) and SQMK-FP cells (SqM kidney epithelial) from fresh-frozen, homogenized liver tissue from animal SBB23. Cells were cultured in Dulbecco’s modified Eagle media (A549 and SqMK-FP) or Roswell Park Memorial Institute Medium (B95a) supplemented with Glutamax (Gibco), 10 % (A549 and B95a) or 5 % (SqMK-FP) FBS, 100 U penicillin ml^–1^ and 100 µg streptomycin ml^–1^. For viral inoculation into cell cultures, approximately 50 mg of liver tissue was homogenized in a 1.5 ml microcentrifuge tube with 450 µl PBS, and centrifuged for 5 min. The supernatant was transferred to a fresh 1.5 ml microcentrifuge tube. The pellet was resuspended in an additional 450 µl PBS, homogenized and centrifuged again, and the supernatants were combined. The supernatant was split into three aliquots of 300 µl, diluted in 2 ml of cell culture media each and filtered through a 0.2 µm syringe filter. The filtered liver homogenate supernatant was inoculated onto 50 % confluent T25 flasks of each cell line and incubated at 37 °C with 5 % CO_2_. After 2 h, an additional 3 ml of medium was added to each flask. Mock inoculation with media only was performed for each cell line. Upon reaching confluency or exhibiting extensive cytopathic effects (CPEs), cell cultures were subjected to two freeze–thaw cycles, centrifuged and filtered as above. Subsequent inoculations were performed with 2 ml undiluted filtered cell culture supernatant into T25 cell culture flasks, or with 5 ml filtered cell culture supernatant diluted with 2 ml media into a T150 cell culture flask. Cell cultures were examined for CPEs by light microscopy over a course of 14 days. To quantify adenovirus propagation, serial log dilutions of cell culture supernatant were tested for the presence of adenoviral DNA by PCR with the Adeno31–Adeno32 primers targeting the 3′ end of the hexon gene.

### Nucleic acid extraction

DNA was extracted from tissue samples using the QIAamp Fast DNA Tissue kit (Qiagen) in accordance with the manufacturer’s instructions. DNA was extracted from clarified cell culture and faecal sample supernatants using the Total Nucleic Acid Extraction Kit with a MagNAPure LC instrument (Roche). Faecal sample supernatants were obtained as follows: faecal samples (approximately 100 µg) were diluted with 900 µl PBS, heated to 85 °C for 10 min, vortexed and centrifuged at 2000 r.p.m. for 5 min.

### Whole genome sequencing

Total nucleic acid extracted from clarified cell culture supernatant was used for deep sequencing. After purification with AMPure XP Beads, a library was prepared using Qiagen’s QIAseq Ultralow Input Library Kit, analysed on an Agilent TapeStation 2200 D1000 assay to determine average size, and quantified using the NEB NGS Library Quantification Kit. The library was normalized to 4 nM, denatured and diluted to approximately 12 pM. A 2 % library of 12.5 pM PhiX was spiked in as an internal control. The library pool was sequenced on an Illumina MiSeq, with a read length of 2×151 bp. Initial base calling and quality scoring were performed with Illumina Real Time Analysis software. Illumina MiSeq Reporter software was used to demultiplex reads, generate FASTQ files, and align indexed reads to the TMAdV genome (HQ913600.1). Alignment was performed within MiSeq Reporter using the Burrows–Wheeler Aligner, which adjusts parameters based on read lengths and error rates, then estimates insert size distribution. Variants were then called in MiSeq Reporter (2.6.3) using the Genome Analysis Toolkit. To generate an initial consensus sequence, Integrative Genome Viewer, with filters set to flag PCR duplicates and to filter out reads with a quality score of less than Q30, was used.

To close the gaps in the initial consensus sequence, specific primers were developed with the aid of DNASTAR SeqBuilder Pro software. To resolve the inverted terminal repeat, primers derived from the TMAdV and SqMAdV-1 MiSeq genome end sequences were used in combination with specific internal primers at each end (Table S2). Sanger sequencing was performed by a commercial vendor (Eurofins Genomics) on PCR products, or, when necessary, on gel-purified PCR products cloned into TOPO TA vectors (Invitrogen). DNASTAR SeqMan Pro software was used to align sequences.

### Identification of structural features

Using DNASTAR MegAlign software, we aligned the annotated genome of TMAdV (HQ913600.1) with the fully sequenced SqMAdV-1 genome. Potential ORFs were then screened in DNASTAR GeneQuest and SeqBuilder Pro to identify the best matches. To verify the accuracy of the ORF sequences, blast x in GenBank was used to align each ORF sequence to Adenoviridae (taxid: 10508) protein sequences.

### PCR-detection of adenoviruses in NWM faecal samples and phylogenetic analysis

Using sequence data from both SqMAdV-1 and TMAdV, we developed nested primer sets to detect sequences in the hexon, IVa2 and DNA polymerase genes (Table S3). In addition we used degenerate nested primers targeting NWM IVa2 sequences [37]. Using SeqMan Pro software, we aligned the retrieved adenovirus sequences, trimmed the primer sequences, and created individual sequence files in SeqBuilder Pro for each positive sample. To create the phylogenetic trees for each of the three targeted areas, we used the clustal w method, which incorporates the unweighted pair group method with arithmetic mean algorithm and neighbourhood joining, within DNASTAR MegAlign software to align the partial adenovirus sequences retrieved from monkey faecal samples with adenovirus sequences from NWM adenoviruses and other primate adenoviruses available in GenBank.

## Results

### Clinical observations of immune-suppressed squirrel monkeys

Six weeks after the second infusion of rituximab, male SBB19 and female SBB20 developed diarrhoea and became weak and lethargic. Supportive therapies were unsuccessful, and SBB19 and SBB20 were humanely euthanized. Shortly after the third infusion of rituximab, SBB22 became severely lethargic with laboured breathing and was humanely euthanized. Three weeks after the third infusion of rituximab, SBB23 was observed to be weak and depressed. Supportive therapy was begun, but SBB23 died later that day. See Table 1 for the treatment timeline and the onset of illnesses described above. The four other immune-suppressed monkeys as well as two non-immune-suppressed monkeys in the study remained in good general health throughout the course of the study and planned infusions of rituximab were cancelled.

### Pathology

**Fig. 2.**
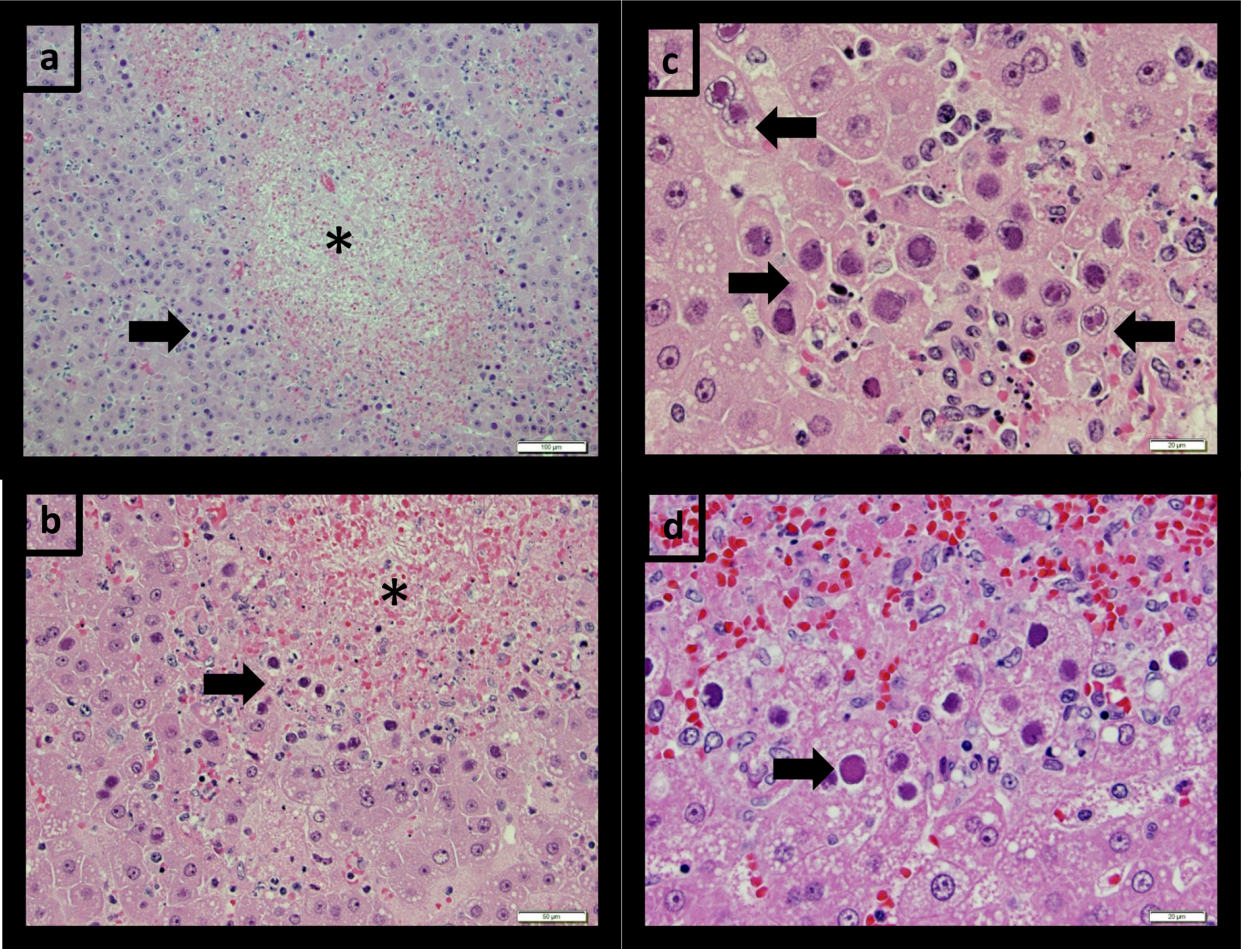
Necrotizing hepatitis with adenoviral inclusions. Animals SBB23 (a–c) and SBB22 (d). Areas of hepatic necrosis are indicated with asterisks and hepatocellular intranuclear inclusions are indicated with arrows. Note, both small eosinophilic and large amphophilic inclusions are present. Haematoxylin and eosin staining. Bars: (a) 100 µm; (b,d) 20 µm; (c) 50 µm.

SBB19 and SBB20 had mild inflammation in the intestinal tract; SBB20 also had interstitial pneumonia, lymphoid depletion in the mesenteric lymph node and spleen, and occasional, presumed viral inclusions in kidney tubular cells. Other monkeys in this dosing group (females SBB17 and SBB18) had mild inflammatory changes in the caecum and colon. At necropsy, findings in SBB23 suggested that a haemorrhagic and/or septic process led to the animal’s death. Microscopic lesions included necrotizing hepatitis and enteritis, lymphoid depletion in several lymph nodes and the spleen, and interstitial pneumonia, as in SBB20. Notably, intranuclear inclusions were present in liver hepatocytes and mucosal epithelial cells in several areas of the small intestine. SBB22 had lesions similar to SBB23; however, inclusions were only observed in the liver (Fig. 2). Other monkeys, including animals with no JCV or rituximab exposure, had no significant findings.

### Testing for potential viral infections

Although the study included inoculation of some animals with JCV, the symptoms, rapid onset and necropsy findings were not suggestive of PML. PCR-based tests of urine and serum samples for JCV DNA in SBB23 were negative prior to the onset of symptoms. Nevertheless, the necropsy findings for animal SBB23 suggested a systemic viral infection. Previous testing within this closed colony has detected the presence of SqMPyV, SaHV-1, SaHV-2 and SaHV-3. Of these four viruses, only SaHV-1 is known to sometimes be pathogenic in its host [38]. Testing of tissues from monkey SBB23 for DNA sequences for each of these viruses, using nested or hemi-nested PCR primer sets, detected the presence of SqMPyV in cerebrum tissue and SaHv-2 in cerebrum and cerebellum tissue. This limited distribution was insufficient to be the cause of a lethal infection and led us to search further for the cause of the lethal infection.

The viral inclusions and multifocal necrotizing hepatitis of SBB23 suggested the possibility of an adenovirus infection [30]; thus, we sought to determine whether adenovirus DNA sequences were present. Initially, we used primers targeting a highly conserved region of the human mastadenovirus hexon gene [34]. Adenovirus sequences were detected in 10 of 10 tissues (cerebellum, cerebrum, kidney, liver, lung, mandibular and mesenteric lymph nodes, medulla-pons, urinary bladder, and spleen) from SBB23. In addition, the mandibular lymph node of SBB19 and liver and spleen tissue of SBB22 were positive in this PCR assay. Sequence data for this short amplicon near the 3′ end of the hexon gene were obtained for the 13 tissues and were identical for all (data not shown). A blast search revealed the greatest similarity to be 91.38 % pairwise identity with simian mastadenovirus WIV19 (accession no. KX505867), an adenovirus isolated from a golden snub-nosed monkey (*Rhinopithecus roxellana*), an OWM endemic to China [4]. Subsequent sequencing of the entire SqMAdV-1 hexon gene showed the greatest similarity to be 83.2 % pairwise nucleotide identity with the TMAdV.

### Cultivation of SqMAdV-1 in SQMK-FP cells

We attempted to propagate the adenovirus in A549 cells, B95a cells and SQMK-FP cells from fresh-frozen homogenized liver tissue from SBB23. Within 3 days, the inoculated SQMK-FP cells exhibited a strong CPE (cell propagation halted, and cells rounded up and detached), while the appearance of the inoculated A549 and B95a cells remained unchanged after 14 days. Secondary infection of 80 % confluent SQMK-FP cells with clarified supernatant from the initial inoculation also led to a strong CPE within 3 days. Testing of serial dilutions of cell culture supernatant by PCR detected the presence of approximately 1e^6^ adenovirus DNA genomes ml^–1^ in the inoculation media for each of the three cell lines. After 14 days, no adenovirus DNA was detected in either the A549 or the B95a cells, while approximately 1e12 genomes ml^–1^ were detected in the SQMK-FP supernatant by limiting dilution PCR. Sequencing of a 2218 bp section of the hexon gene, including the hypervariable region, amplified from supernatant samples collected on 0, 7, 14 and 20 days post-infection from the SQMK-FP cell cultures revealed no changes in the SqMAdV-1 sequence over time. Thus, propagation of the SqMAdV-1 was successful in the SQMK-FP cells, but not in the A549 or B95a cell lines.

### Whole-genome sequencing

**Fig. 3.**
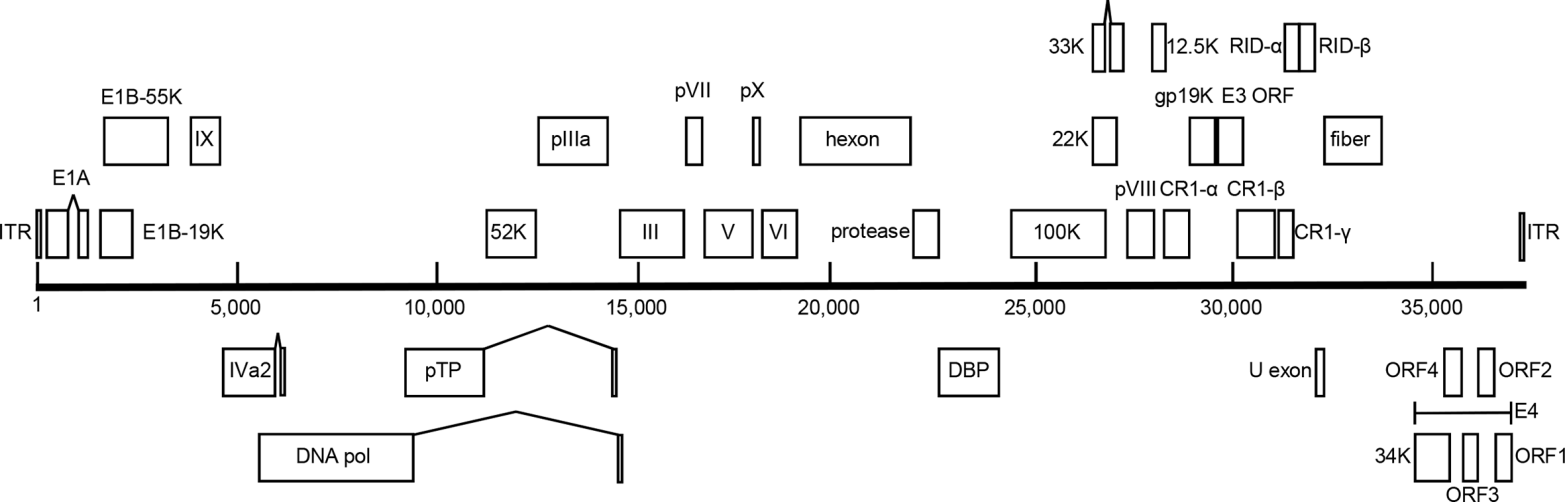
Genome organization of SqMAdV-1. Predicted protein coding regions and the inverted terminal repeat regions are shown as boxes. ORFs encoded on the forward strand are above the line, and ORFs encoded on the reverse strand are below the line. The scale indicates nucleotide position along the genome.

Using DNA extracted from clarified cell culture supernatant, whole-genome sequencing was begun with MiSeq sequencing aligned to the TMAdV genome (accession no. HQ913600.1). Of the 54 417 610 paired-end reads produced, 12 412 609 aligned to TMAdV. From these reads, a consensus sequence of 36 838 bp was generated. Due to discordance between the TMAdV reference sequence and the new SqMAdV-1 sequence, a large portion of bases (more than 10 000) could not be called. Specific primers were designed from this consensus sequence to amplify and sequence (by Sanger method) the ambiguous DNA in sections across the entire genome. In an iterative fashion, as the ambiguous sections of the genomes were successfully sequenced, additional primers were designed to amplify and sequence the remaining sections of DNA. Ultimately, the genome was found to be 37 431 bp in length, with a G+C content of 61.6 %. A comparison of the entire SqMAdV-1 genome with other primate adenovirus genomes showed it to have greatest similarity to the TMAdV, with a nucleotide identity of 80.5 %. The genomic structure of the SqMAdV-1 was deduced through comparison with the TMAdV and other primate mastadenoviruses, and found to contain 36 ORFs (Fig. 3). Two features distinguish the SqMAdV-1 genomic structure from other primate adenoviruses: (1) within the E3 region there are two ORFs between CR-1α and CR-1β, the first of which is putatively gp19K, while the function of the second ORF is unknown (a blast search identified no similarities); and (2) the DNA polymerase gene, at 3948 bp, is approximately 10 % longer than the DNA polymerase genes of other primate adenoviruses.

Alignment of SqMAdV-1 with TMAdV and other primate adenoviruses revealed that the unusually long length of the DNA polymerase gene is due to a nucleotide polymorphism at the expected stop codon location (Fig. S1). A protein blast search of the 1315 aa sequence for SqMAdV-1 DNA polymerase produced alignments with a maximum query coverage of 90 %, indicating that no other known primate adenoviruses have a similar polymorphism. To verify that the stop codon mutation was not an artefact of cell culture, we amplified and sequenced DNA extracted from liver tissue of SBB23 across this area of the genome and found that it was identical to that of the DNA extracted from the cell culture supernatant.

### Discovery of additional adenoviruses in archived faecal samples

Archived faecal samples from NWMs housed at the KCCMR were screened using nested PCR primer sets targeting SqMAdV-1 sequences (Table S3), and degenerate nested primers targeting NWM IVa2 sequences [37]. The specimens we tested were collected from immune-suppressed Bolivian squirrel monkeys, non-immune-suppressed Bolivian, Peruvian and common squirrel monkeys, and from owl monkeys (Table 2). We targeted three regions of interest: (1) the region surrounding the polymorphism at the expected DNA polymerase stop codon; (2) a conserved region of the IVa2 gene to enable comparison with NWM adenovirus sequences recently reported by Padgorski *et al*. [11] and (3) the hypervariable region of the hexon gene, as this region is important for the classification of adenoviruses [39]. While no adenovirus sequences were detected in the 13 owl monkey faecal samples tested, in the 95 squirrel monkey specimens tested, adenovirus sequences were detected in 37.

**Table 2.** Faecal samples PCR-tested for adenovirus sequences

Host species	Age	Collection dates	Immune-suppressed	No. of monkeys	No. of samples	No. of monkeys with positive samples	No. of positive samples
Bolivian squirrel monkey *Saimiri boliviensis boliviensis*	12–18 months	2009	no	4	16	4	8
	2–3 years	2017–18	yes	18	19	8	9
			no	4	4	1	1
	7–10 months	2017–18	yes	5	5	5	5
			no	2	2	2	2
Peruvian squirrel monkey	>3 years	2010	no	39	39	10	10
*Saimiri boliviensis peruviensis*							
Common squirrel monkey	*>*3 years	2009	no	10	10	2	2
*Saimiri sciureus*							
Nancy Mae's owl monkey *Aotus nancymaae*	>3** **years	2009	no	13	13	0	0
Total				95	108	32	37

Among the 37 adenovirus-positive faecal specimens, 54 unique sequences were detected across the three targeted genomic areas (Table 3). Because the distance between the IVa2-DNA polymerase sequences and the hexon sequences is so large, we did not attempt to amplify the entire area between these regions, and thus it was not possible to determine the exact number of different adenoviruses detected. Phylogenetic analysis showed that these squirrel monkey adenovirus sequences clustered primarily into two groups, and our numbering scheme reflects this by grouping the detected sequences from the three amplified areas by similarity with SqMAdV-1 (SqMAdV-1.#), and similarity with TMAdV (SqMAdV-2.#). The third adenovirus species detected was designated SqMAdV-3; the sequences detected in the positive samples from five monkeys were identical to each other.

**Table 3.** Novel adenoviruses detected by PCR

Host species	Proposed adenovirus name	Host	Collection date^a^	PCR results^b^		
				Hexon	IVa2	IVa2 – DNA pol
Bolivian squirrel monkey *Saimiri boliviensis boliviensis*	Squirrel monkey adenovirus-1.0	SBB23	12/15/2017c	+	+	+
		SBB22	11/22/2017	+	+	+
	Squirrel monkey adenovirus-1.1	SBB02	3/2 and 3/30/2009	**−**	+	+
	Squirrel monkey adenovirus-1.2	SBB04	3/9/2009	−	+	+ (1.0)
		SBB03	4/13/2009	−	+	−
	Squirrel monkey adenovirus-1.3	SBB03	4/27/2009	+	+	**−**
	Squirrel monkey adenovirus-1.4	SBB01	5/11/2009	+	+	**+ (1.1)**
		SBB04	5/11/2009	**−**	+	**+ (1.1)**
		SBB14	2/24/2018	**−**	+	**−**
	Squirrel monkey adenovirus-1.5	SBB11	12/30/2017	**+**	−	−
	Squirrel monkey adenovirus-1.6	SBB08	2/28/2018	−	−	**+**
	Squirrel monkey adenovirus-1.7	SBB12	2/28/2018	**+**	**+ (1.1**)	**+**
	Squirrel monkey adenovirus-1.8	SBB07	3/16/2018	**+**	−	−
	Squirrel monkey adenovirus-1.9	SBB15	2/24/2018	**+**	**+**	**+**
	Squirrel monkey adenovirus-2.0	SBB02	3/2/2009	**+**	−	**+**
	Squirrel monkey adenovirus-2.1	SBB02	3/9/2009	**+**	**+**	**+**
	Squirrel monkey adenovirus-2.2	SBB04	3/9/2009	**+**	−	**+**
		SBB03	4/27/2009	**+**	−	**+**
	Squirrel monkey adenovirus-2.3	SBB01	3/23/2009	**+**	**+**	**+ (2.1**)
	Squirrel monkey adenovirus-2.4	SBB01	5/11/2009	**+**	−	**+**
	Squirrel monkey adenovirus-2.5	SBB05	12/16/2017	**+**	**+**	**+ (2.2**)
		SBB10	3/6/2018	−	**+**	**+ (2.2**)
	Squirrel monkey adenovirus-2.6	SBB06	2/28/2018	**+**	**+ (2.3**)	**+ (2.2**)
	Squirrel monkey adenovirus-2.7	SBB07	3/16/2018	−	**+**	**+**
		SBB13	2/28/2018	−	−	**+**
	Squirrel monkey adenovirus-2.8	SBB09	3/2/2018	**+**	**+ (2.3**)	**+ (2.2**)
		SBB11	12/30/2017	−	**+ (2.3**)	**+ (2.2**)
	Squirrel monkey adenovirus-2.9	SBB12	2/28/2018	**+**	−	−
	Squirrel monkey adenovirus-2.10	SBB13	3/21/2018	**+**	**+ (2.3**)	**+ (2.2**)
	Squirrel monkey adenovirus-2.11	SBB14	2/24/2018	**+**	−	−
	Squirrel monkey adenovirus-2.12	SBB16	2/24/2018	**+**	**+**	**+**
		SBB18	2/24/2018	**+**	**+**	**+**
		SBB21	2/24/2018	**+**	**+**	**+**
	Squirrel monkey adenovirus-2.13	SBB17	2/24/2018	**+**	**+ (2.3**)	**+ (2.0**)
	Squirrel monkey adenovirus-3	SBB14	2/24/2018	−	**+**	**+**
		SBB17	2/24/2018	−	−	**+**
Peruvian squirrel monkey *Saimiri boliviensis peruviensis*	Squirrel monkey adenovirus-1.10	SBP07	10/13/2010	−	−	**+**
	Squirrel monkey adenovirus-1.11	SBP10	10/13/2010	−	**+**	−
	Squirrel monkey adenovirus-2.14	SBP01	10/13/2010	**+ (2.12**)	**+**	**+ (2.2**)
		SBP06	10/14/2010	−	**+**	**+ (2.2**)
	Squirrel monkey adenovirus-2.15	SBP02	10/13/2010	**+**	**+ (2.7**)	**+**
		SBP09	10/7/2010	**+**	**+**	−
		SBP08	10/7/2010	−	**+**	**+**
		SBP03	10/7/2010	−	**+**	−
	Squirrel monkey adenovirus-3	SBP04	10/16/2010	**+**	**+**	**+**
		SBP05	10/7/2010	−	−	**+**
		SBP07	10/13/2010	**+**	**+**	**+**
Common squirrel monkey *Saimiri sciureus*	Squirrel monkey adenovirus-2.16	SS01	5/7/2009	**+**	**+**	**+**
	Squirrel monkey adenovirus-2.7	SS02	5/7/2009	−	**+ (2.7**)	−

a, All specimens are fecal unless otherwise noted. b, Liver tissue. c, “−” negative, “+” positive, “#.#” sequence is identical to the specified adenovirus in the amplified region.

Color code for species of adenovirus detected in different specimens:
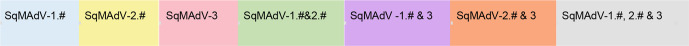

The overall prevalence of adenoviruses detected in the squirrel monkey faecal specimens was 39 %. Prevalence was highest for SqMAdV-2 species variants with 28 of 95 (29 %) squirrel monkey samples testing positive and lowest for the SqMAdV-3 species with five of 95 of the specimens testing positive. With few exceptions, nested PCR was required for the detection of adenovirus sequences, indicating that most samples contained low titres of adenovirus. For the monkeys 1–3 years of age, the prevalence of adenoviruses detected in faecal samples was 46 %, and was not significantly different between immune-suppressed and non-immune-suppressed animals (nine of 19 and nine of 20, respectively). The prevalence of adenoviruses detected in specimens from monkeys younger than 1 year old was 100 %. However, the number of specimens for this group was small, with five from immune-suppressed monkeys and two from non-immune suppressed monkeys. At 24 %, the lowest prevalence of adenovirus detected was in faecal specimens from monkeys 3 years of age and older.

### Phylogenetic analysis

**Fig. 4.**
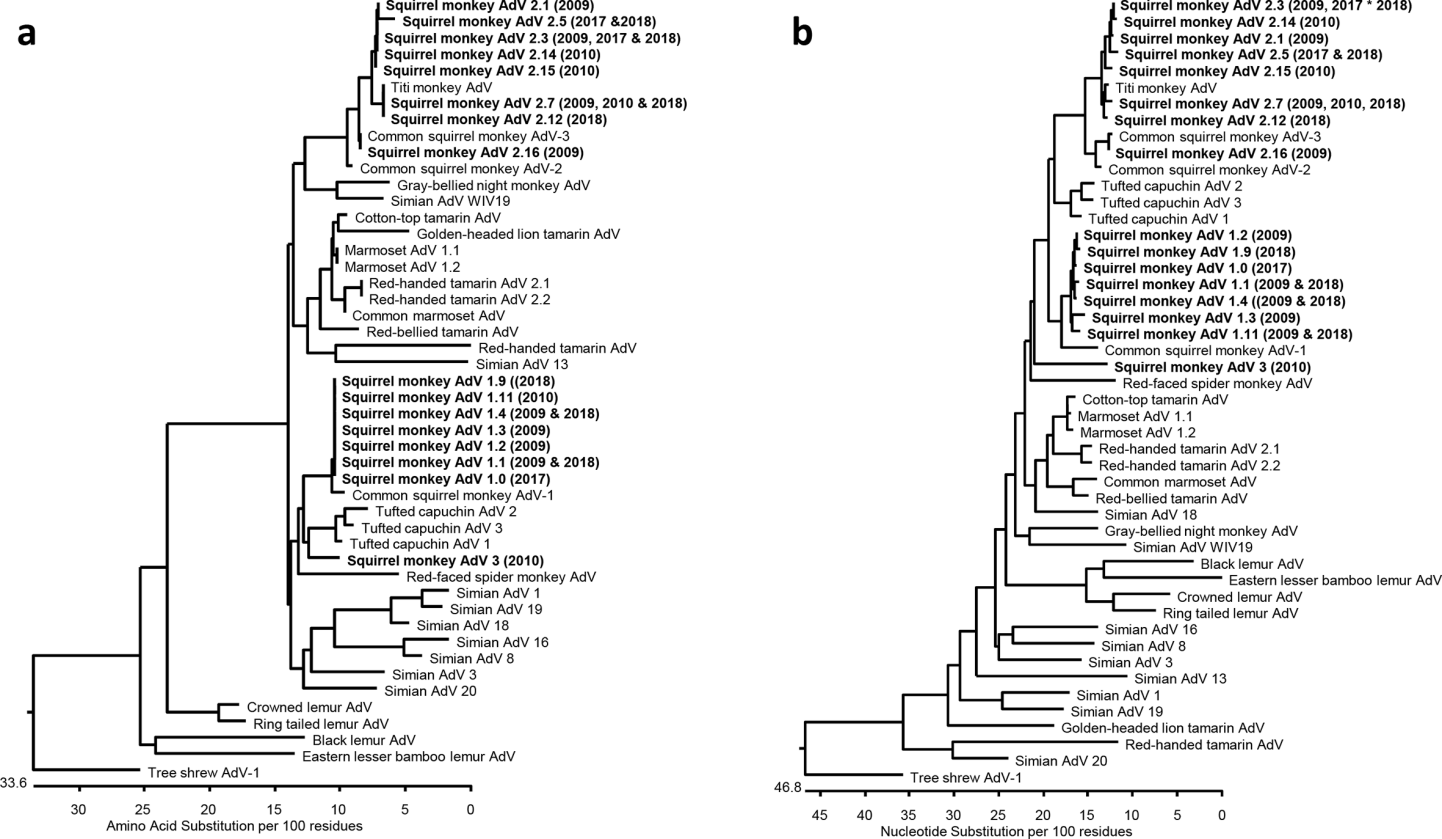
Phylogeny of adenoviruses detected in this study. Comparison of squirrel monkey adenoviruses to adenoviruses of NWMs, other primates and the tree shrew, based on partial sequences of the IVa2 gene. Adenoviruses identified in this report are in bold type, and the year(s) of collection for each are provided here and in Table 2. Accession numbers of comparison sequences are listed in Table S4. (a) Comparison based on 84 aa. (b) Comparison based on 252 nt.

Phylogenetic analysis of the three SqMAdV genomic regions revealed that there are at least three separate SqMAdV species. Within the IVa2 gene, the area of the adenovirus genome for which the most sequence data for NWM adenoviruses is available, the SqMAdV-1 cluster of adenoviruses is most closely related to the common squirrel monkey AdV-1, detected by Podgorski in a faecal specimen from a Hungarian zoo [11], while the SqMAdV-2 cluster of adenoviruses is most closely related to the common squirrel monkey AdV-2 and -3 and with TMAdV (Fig. 4). Notably, the SqMAdV-1 variants and SqMAdV-3 have higher pairwise amino acid identities with the tufted capuchin AdVs1–3 (93–96 %) than to the SqMAdV-2 cluster of adenoviruses. (Fig. S2).

The high similarity of SqMAdV-1 to common squirrel monkey AdV-1 within the IVa2 gene provided an opportunity to determine whether the DNA polymerase stop codon mutation common to all the SqMAdV-1 variants we detected in *S. boliviensis* faecal specimens was also present in the adenovirus detected in the *S. sciureus* faecal specimen that was collected in a Hungarian zoo. Podgorski and colleagues generously assisted, successfully amplifying and sequencing the region surrounding the DNA polymerase stop codon mutation site, revealing that the common squirrel monkey AdV-1 also contains this unusual mutation (GenBank accession no. MN695333).

Phylogenetic analysis of the 3′ end of the DNA polymerase gene and the hexon hypervariable region produced phylogenetic trees similar to those based on the partial IVa2 sequence with respect to the relationship of the three SqMAdV species and other primate adenoviruses (Fig. S3).

## Discussion

Using primers targeting a highly conserved region of the human adenovirus hexon gene [34], we identified a novel adenovirus as the cause of fatal hepatitis and pneumonia in a young immune-suppressed squirrel monkey. Subsequent analysis of tissues from the index case revealed that all tissues tested were positive for this novel adenovirus. In addition, three tissue samples from two other study animals tested positive.

We surmised that the detected adenovirus DNA originated from either a previously unknown adenovirus endemic to squirrel monkeys or potentially from a human adenovirus transmitted to the monkeys from researchers and technicians in contact with the squirrel monkey colony. Less likely, but still a possibility, was a cross-species transmission of an adenovirus from another primate colony at the KCCMR facility. At the time of the adenovirus infections in the squirrel monkeys, the other primates housed at the KCCMR included two additional species of squirrel monkeys (*S. boliviensis peruviensis* and *S. sciureus),* owl monkeys (*Aotus nancymaae),* rhesus macaques (*Macaca mulatta*) and chimpanzees (*Pan troglodytes*).

To identify the mostly likely source of the adenovirus infection in the squirrel monkeys, we obtained sequence data for the short amplicon in the hexon gene for the 13 adenovirus-positive squirrel monkey tissues. The sequence was identical for all tissues, and was found to have greatest similarity to SAdV-WIV19. SAdV-WIV19 was isolated from a faecal sample from a wild golden snub-nosed monkey (*Rhinopithecus roxellana*), an OWM of the subfamily Colobinae. Reported in 2016, SAdV-WIV19 is the only adenovirus from the family Colobinae to be fully sequenced, and phylogenetic analysis shows it to be distantly related to other OWM adenoviruses [4]. Other than the SAdVWIV19, the short adenovirus hexon gene sequence from the squirrel monkey tissues shared the highest pairwise identity (89%) with human adenovirus D variants. These results suggested that the source of the adenovirus infection in the squirrel monkeys was a novel adenovirus. Using degenerate primers targeting the 5′ end of the adenovirus hexon gene we obtained additional sequence data, which revealed its similarity to the TMAdV, which was previously the only fully sequenced adenovirus from an NWM. The similarity to TMAdV hinted that SqMAdV-1 was an NWM adenovirus, although the dearth of published sequence information for NWM adenoviruses made it impossible to immediately ascertain whether squirrel monkeys are the natural hosts of SqMAdV-1.

To gain further understanding of the epidemiology of SqMAdV-1, we tested archived faecal samples from each of the four species of NWMs housed in the KCCMR (*S. boliviensis bolliviensis*, *S. boliviensis peruviensis*, *S. sciureus* and *Aotus nancymaae*) for SqMAdV-1 adenovirus sequences. We did not detect any adenovirus sequences in the 13 owl monkey faecal specimens tested, but numerous samples from each of the three *Saimiri* species were positive for adenovirus sequences. We detected not only a cluster of several adenoviruses with high similarity to SqMAdV-1, but another larger cluster of adenoviruses with strikingly high similarity to TMAdV, and one adenovirus, SqMAdV-3, with less similarity (72–88 % in the three targeted genomic areas) to either SqMAdV-1 or TMAdV. Adenoviruses from both SqMAdV-1 and SqMAdV-2 clusters were detected in Bolivian squirrel monkey samples collected in 2009–10 and in 2017–18 indicating that both have likely been continuously circulating in the colony. SqMAdV-3 was detected in only five of 95 squirrel monkey samples tested, with three positive samples collected from Peruvian squirrel monkeys in 2010 and two positive samples collected from Bolivian squirrel monkeys in 2018, potentially indicating a lower incidence of this infection. In 11 samples, more than one adenovirus was detected, and one specimen contained sequences from all three SqM adenovirus species; the significance of this is unclear. The prevalence of adenoviruses in the squirrel monkey faecal samples was 54 % for Bolivian squirrel monkey samples, 26 % for Peruvian squirrel monkey samples and 20 % for common squirrel monkeys. The higher prevalence of adenoviruses detected in the Bolivian squirrel monkey samples may be due to the younger ages of these monkeys.

Phylogenetic analysis for the three targeted genomic areas produced consistent results with respect to the relationship between the three SqMAdV species. Comparison of the IVa2 sequences obtained from our samples with the 20 NWM partial IVa2 adenovirus sequences available in GenBank revealed that SqMAdV-1 variants are most similar to common squirrel monkey AdV-1 (98.8 % pairwise amino acid identity), SqMAdV-2 variants are most similar to common squirrel monkey AdV-3 and TMAdV (97.6–100% pairwise amino acid identity) and SqMAdV-3 is most similar to tufted capuchin AdV-3 (96.4 % pairwise amino acid identity). The high similarity between squirrel monkey and tufted capuchin adenoviruses is not surprising, as they are closely related phylogenetically; and, in the wild, squirrel monkeys and capuchin monkeys have largely overlapping geographical ranges, and are even known to share trees [40, 41]. Separately, the discovery of the SqMAdV-2 cluster of adenoviruses, an apparently endemic virus in squirrel monkeys with high similarity to TMAdV, suggests that the deadly pneumonia outbreak in the titi monkey colony at the CNPRC in 2009 may have been caused by an adenovirus originating in *Saimiri* species. This theory is supported by the fact that common squirrel monkeys (*S. sciureus*) were housed at the CNPRC within the decade prior to the outbreak in titi monkeys [42, 43].

The discovery of three different adenovirus species in Bolivian and Peruvian squirrel monkeys (*S. boliviensis*) expands our knowledge of adenovirus diversity and epidemiology in NWMs. In particular, the full genome sequencing and characterization of SqMAdV-1, with its unique DNA polymerase gene, provides an intriguing opportunity for new discoveries. Additional investigation will be necessary to determine: (1) whether there are SqMAdV-1 variants endemic in additional NWM species; (2) whether any other NWM adenovirus species contain a DNA polymerase gene of similar length due to a stop codon mutation; and (3) whether the mutation is associated with enhanced replication and/or pathogenesis. Of equal importance, these discoveries highlight the necessity for monitoring laboratory animals for the presence of adenoviruses, especially young and immunosuppressed animals, due to their vulnerability to fatal infection. In addition, the potential for cross-species transmission remains a concern as some adenoviruses are highly infectious.

## Supplementary Data

Supplementary material 1
