# Supplementary material for: Epidemiological and molecular characterization of a novel adenovirus of squirrel monkeys after fatal infection during immunosuppression
Source: Microb Genom. 2020 Jul 2;6(9):mgen000395. doi: 10.1099/mgen.0.000395 (PMC7643968; doi:10.1099/mgen.0.000395)
Supplement: Supplementary material 1 [file mgen-6-395-s001.pdf]

Table S1. Primers used for initial virologic assessments

Virus designation	GenBank accession #	genome target	primer name	primer sequence	amplicon size	annealing temperature
SaHV-1<br>Herpes tamarinus	HM625781.1	helicase	HeliF<br>HeliR	TGG CCA GCC GAG CCA TCT TTG<br>GAT GCC GCA CAG GAA ATC CAC CA	698	61
			HeliF3<br>HeliR3	GCA GAG CGC GAG CGG TAC<br>CAC TTG GTG TAA GTA CCG CAT AGT CGG	651	61
SaHV-2<br>herpesvirus saimiri	M31122.1	glycoprotein B	HVS-651F<br>HVS-1151R	TAC TTT GTG ACT GCG CTT GG<br>TGC TAT TAA TGG CTG CCA CA	501	60
			HVS-1142R*	TGG CTG CCA CAC TAA AAT CA	492	60
SaHV-3<br>squirrel monkey lymphocryptovirus	JF692217.1	DNA polymerase	SaHV-3 Fout<br>SaHV-3 Rout	CAG GCR CAC AAR CTC TGT TAT TC<br>CCC TCG GGC GCG AGT CC	461	60
			SaHV-3 Fin<br>SaHV-3 Rin	CCG GGT AYT GAG TGT AGA CTG CG<br>CCC AAG CTC GAC SCC TCA G	410	60
SqMPyV<br>squirrel monkey polyomavirus	NC009951.1	regulatory region	4921F<br>489F	GCG GTT TGC TCA TCT CCA CCT TTG TCT GG<br>GAG AGG GAA ATT CCC GTA CTG CTC C	851	61
			696R<br>653R	GCC ACA CTG ATG TCA TGC CGA ATG TAG C<br>GGC ACG ACT TTC AGT TAT CCT CGG	740	61

Thermocycling conditions: initial denaturation for 5 minutes at 94 ºC; amplification for 40 cycles: denaturation - 30 seconds at 94 ºC , anneal - 30 seconds at temperature shown in table, extension - 30 seconds at 72 ºC; final extension for 7 minutes at 72 ºC.

*Pairs with primer HVS-651F for hemi-nested PCR.

Table S2. Primers used to resolve Inverted terminal repeat

	primer name	primer sequence
genome end primers	ITR-1F	CCA TCA TCA ATA ATA CCT CAA AAA CG
	ITR-TMAdV	CCA TCA TCA ATA ATA TAC CTC AA
internal primers near 5' end	371R	GCC CGG CAA TAA AAC CAC ACC
	423R	CTC TTC TCG CGG GGC ACT CA
	467R	TCA GCT GAC TCC GCG GTG CCC
	507R	CAG CAC TTC CTG GAT CAG TTC CAG
internal primers near 3' end	36882F	AGA AAA TGG CGA TCG CTG ACT GA
	36588F	CCG TGA GCT CGC TCG GCT G
	37034F	CGC GCC AAA ACA CCG ACT TC

Thermocycling conditions: initial denaturation for 5 minutes at 94 ºC; amplification for 40 cycles: denaturation - 30 seconds at 94 ºC, anneal - 30 seconds at 60 ºC, extension - 30 seconds at 72 ºC; final extension for 7 minutes at 72 ºC.

Table S3. Primers used to screen fecal samples for adenovirus sequences

genome target	primer name	primer sequence	amplicon size	annealing temperature
Hexon hypervariable region	Hex Fout	ACA TGG CCA GCA CKT ACT TTG ACA	854	59
	Hex Rout	CCT CAC GTC GGG ATC RTA ACT GTC		
	Hex F2	GGR CCC AGC TTC AAR CCC TAC AG	757	59
	Hex R2	GSG TTC TRT CAC YCA RCG CAT C		
IVa2	IVa2 Fout	CGC GGG GAT GSA GGT AGA GC	748	61
	IVa2 Rout	AAY TAC AMC MTG CAA CCC GTC ATC		
	IVa2 Fin	ATG CGG GGG CTG ATG AKR TG	509	61
	IVa2 Rin	CAG CTC CTC CGC AAC CGC ATG T		
DNA polymerase stop codon	DPol Fout	AGG TTG CGG AGG AGC TGT GAC TT	545	61
	DPol Rout	CGC CCG CTW CGA GAC CAG T		
	DPol Fin	GAT GAC GGG TTG CAG GGT GTA GTT	333	61
	DPol Rin	GCA TCG CCT CGT CCC CTA CTC		

Thermocycling conditions: initial denaturation for 5 minutes at 94 ºC; amplification for 40 cycles: denaturation - 30 seconds at 94 ºC , anneal - 30 seconds at temperature shown in table, extension - 30 seconds at 72 ºC; final extension for 7 minutes at 72 ºC.

Table S4. Accession #s of adenovirus sequences used in phylogenetic trees

Adenovirus name	Accession #
Tree shrew AdV-1	AC_000190
Crowned lemur adenovirus	MG574566
Black lemur adenovirus 1	MG574567
Eastern lesser bamboo lemur adenovirus	MG574574
Ring tailed lemur adenovirus	MG574575
Gray-bellied night monkey adenovirus	MG574577
Red-faced spider monkey adenovirus	MG574578
Marmoset adenovirus 1.1	MG574579
Marmoset adenovirus 1.2	MG574580
Common marmoset adenovirus	MG574581
Tufted capuchin adenovirus 1	MG574582
Tufted capuchin adenovirus 2	MG574583
Tufted capuchin adenovirus 3	MG574584
Golden-headed lion tamarin adenovirus	MG574585
Red-bellied tamarin adenovirus	MG574586
Red-handed tamarin adenovirus 1	MG574587
Red-handed tamarin adenovirus 2.1	MG574588
Red-handed tamarin adenovirus 2.2	MG574589
Cotton-top tamarin adenovirus	MG574590
Common squirrel monkey adenovirus 1	MG574591
Common squirrel monkey adenovirus 2	MG574592
Common squirrel monkey adenovirus 3	MG574593
Simian AdV 1	NC_006879
Simian AdV 3	NC_006144
Simian AdV 8	NC_28113
Simian AdV 13	NC_028103
Simian AdV 16	NC_028105
Simian AdV 18	NC_022266
Simian AdV 20	NC_020485
Simian AdV 21	AC_000010
Simian AdV 25	AC_000011
Simian WIV19	KX505867

Human AdV F NC_001454	CCAAAGCCGTCCCAACCCCCGCAACCAAGAAGTTTGTTGGATCGAGATGCCA TAG---ACCATATTACCGAGCTCTGGGA
Human AdV 1 NC_000017	CGAAAGCCGCCCCAACCCGCGAAACGAGGAGATATGCTGGATCGAGATGCCG TAG---AGCACGTGACCGAGCTGTGGGA
Human AdV 7 AC_000018	CAGGAGCCGTCCCAACCCGCGAAACGAGGAGGTCTGCTGGATCGAGATGCCA TAG---AGCACATCACCGAGCTCTGGGA
Human AdV 35 AC_000019	CAGGAGCCGTCCAAACCCGCGAAACGAGGAGGTCTGCTGGATCGAGATGCCA TAG---AACACATCACTGAACTGTGGAA
Human AdV 52 DQ923122	CGAAAGTCGGCCCAACCCCCGCAACCAAGAAGTCTGCTGGATCGAGATTCCA TAG---AGCACGTCACCGAGCTCTGGGA
Human AdV A NC_001460	CAACAGCCAGCCAAACCCCCGCAACGAGGAAGTTTGCTGGATCGAGATGGCA TAG---AAAATATAACTGAGCTATGGGA
Human AdV D NC_010956	TCGCAGTCGTCCCAACCCCCGAAACGAGGAAGTCTGCTGGATCGAGATGCCG TAG---AGCACGTCACCGAGCTCTGGGA
Human AdV E NC_003266	CCGCAGTCGCCCCAACCCGCGAAACGAGGAGGTCTGCTGGATCGAGATGCCG TAG---AGCACATCACCGAGCTCTGGGA
Simian AdV WIV19 KX505867	CAGGAGCAGACCCAACCCCCGCAACGAGGAAGTCTGCTGGATGGAGATGCCT TAG---AGCACATTACCGAGCTCTGGGA
Simian AdV 1 NC_006879	CGAAAGTCGGCCCAACCCCCGAAACCAAGAAGTCTGCTGGATCGAGATTCCA TAG---AGCACGTCACCGAGCTCTGGGA
Simian AdV 3 NC_006144	CCAGAGCCGCCCCAACCCCCGAAACGAGGAGGTCTGCTGGATCGAGATGCCG TAG---AGCACGTAACCGAGCTCTGGGA
Simian AdV 8 NC_28113	CGAAAGCCGCCCCAATCCCCGCAACCAGGAAATCTGCTGGATCGAGATGCCT TAGTGGAGGACGTGACCGAACTCTGGGA
Simian AdV 13 NC_28103	CAACAGCCAACCCAACCCAAGAAACCAGGAAATCTACTGGATCGAGATGCCG TAG---AACACGTCACAGAACTCTGGGA
Simian AdV 16 NC_028105	CGAAAGCCGCCCCAACCCTCGCAACCAAGAGACTTGCTGGATCGAGATGCCC TAG---AGCACGTCACCGAGCTCTGGGA
Simian AdV 18 NC_022266	CCAAAGCCGCCCCAACCCCCGCAACCAAGAAGTCTGCTGGATCGAGATGCCG TAG---AACACGTCACCGAGCTCTGGGA
Simian AdV 19 NC_028107	CGAAAGTCGTCCCAACCCTCGCAACCAAGAAGTCTGCTGGATCGAGATGCCA TAG---AACACGTTACCGAACTGTGGGA
Simian AdV 20 NC_020485	CCAGAGCCGACCCAACCCCAGAAACGAGGAAGTGTGTTGGATCGAGATGCTG TAG---AGCATATCACAGAACTTTGGGA
Simian AdV 21 AC_000010	CAGGAGCCGTCCCAACCCGCGAAACGAGGAGGTCTGCTGGATCGAGATGCCA TAG---AACACATTACCGAGCTCTGGGA
Simian AdV 25 AC_000011	CCGCAGCCGTCCCAACCCGCGAAACGAGGAGGTCTGCTGGATCGAGATGCCG TAG---AGCACATCACCGAGCTCTGGGA
Titi Monkey AdV HQ913600	CAACAGCCATCCCAACCCAAGAAACCAAGAAACCTGCTGGATCGAGATGCCC TAG---AACACGTCCGCGAGATCTGGGA
**Squirrel Monkey AdV 1.0**	CAACAGCCGACCCAATCCAAGAAACCAAGAAACCTGCTGGATCGAGATGCCC **TCG**---AGCACGTCCGAGAGATCTGGGA

DNA polymerase

Figure S1. Alignment of SqM AdV-1 with TMAdV and other human and simian adenoviruses at the 3' end of the DNA polymerase gene

Percent Identity

	1	2	3	4	5	6	7	8	9	10	11	12	13	14	15	16	17	18	19	20	21	22	23	24	25	26	27	28	29	30	31	32	33	34	35	36	37	38	39	40	41	42	43	44	45	46	47	48		
**1**		75.0	78.6	75.0	73.8	75.0	83.3	81.0	71.4	76.2	76.2	76.2	75.0	75.0	73.8	73.8	69.0	84.5	75.0	72.6	77.4	76.2	75.0	73.8	78.6	77.4	76.2	71.4	77.4	75.0	72.6	73.8	77.4	77.4	77.4	77.4	77.4	77.4	77.4	72.6	72.6	72.6	71.4	71.4	72.6	72.6	73.8	75.0	**1**	Black lemur AdV
**2**	30.4		92.9	90.5	89.3	97.6	84.5	76.2	92.9	91.7	98.8	98.8	95.2	89.3	98.8	98.8	86.9	82.1	86.9	84.5	83.3	88.1	89.3	89.3	91.7	85.7	90.5	89.3	71.4	94.0	94.0	94.0	91.7	91.7	91.7	91.7	91.7	91.7	91.7	88.1	88.1	86.9	89.3	89.3	88.1	88.1	89.3	92.9	**2**	Common marmoset AdV
**3**	25.3	7.5		91.7	90.5	92.9	85.7	77.4	89.3	89.3	94.0	94.0	92.9	89.3	91.7	91.7	83.3	85.7	86.9	85.7	88.1	90.5	86.9	90.5	90.5	89.3	90.5	88.1	72.6	94.0	91.7	92.9	98.8	98.8	98.8	98.8	98.8	98.8	98.8	89.3	89.3	88.1	88.1	88.1	89.3	89.3	90.5	94.0	**3**	Common squirrel monkey AdV-1
**4**	30.4	10.2	8.9		98.8	90.5	82.1	76.2	88.1	88.1	91.7	91.7	89.3	86.9	89.3	89.3	81.0	82.1	82.1	84.5	82.1	85.7	82.1	89.3	88.1	84.5	91.7	96.4	72.6	94.0	91.7	91.7	92.9	92.9	92.9	92.9	92.9	92.9	92.9	97.6	97.6	96.4	96.4	96.4	97.6	97.6	98.8	90.5	**4**	Common squirrel monkey AdV-2
**5**	32.2	11.6	10.2	1.2		91.7	81.0	75.0	88.1	89.3	90.5	90.5	88.1	85.7	88.1	88.1	82.1	81.0	81.0	84.5	81.0	84.5	81.0	90.5	86.9	83.3	92.9	97.6	73.8	92.9	90.5	90.5	91.7	91.7	91.7	91.7	91.7	91.7	91.7	98.8	98.8	97.6	97.6	97.6	98.8	98.8	100.0	91.7	**5**	Common squirrel monkey AdV-3
**6**	30.4	2.4	7.5	10.2	8.9		84.5	76.2	94.0	91.7	98.8	98.8	95.2	86.9	96.4	96.4	86.9	82.1	84.5	85.7	83.3	88.1	86.9	91.7	89.3	85.7	92.9	89.3	73.8	94.0	91.7	91.7	91.7	91.7	91.7	91.7	91.7	91.7	91.7	90.5	90.5	89.3	89.3	89.3	90.5	90.5	91.7	92.9	**6**	Cotton-top tamarin AdV
**7**	18.9	17.4	15.9	20.5	22.0	17.4		85.7	79.8	79.8	85.7	85.7	84.5	77.4	83.3	83.3	73.8	96.4	79.8	75.0	77.4	82.1	82.1	78.6	78.6	77.4	82.1	78.6	76.2	83.3	81.0	82.1	84.5	84.5	84.5	84.5	84.5	84.5	84.5	79.8	79.8	79.8	78.6	78.6	79.8	79.8	81.0	83.3	**7**	Crowned lemur AdV
**8**	22.0	28.7	27.0	28.7	30.4	28.7	15.9		71.4	71.4	77.4	77.4	77.4	72.6	75.0	75.0	72.6	84.5	75.0	69.0	72.6	75.0	76.2	73.8	73.8	72.6	73.8	73.8	75.0	77.4	75.0	76.2	78.6	78.6	78.6	78.6	78.6	78.6	78.6	75.0	75.0	75.0	73.8	73.8	75.0	75.0	75.0	77.4	**8**	Eastern lesser bamboo lemur AdV
**9**	36.0	7.5	11.6	13.0	13.0	6.2	23.6	36.0		86.9	94.0	94.0	90.5	83.3	92.9	92.9	82.1	77.4	81.0	84.5	79.8	84.5	83.3	86.9	85.7	82.1	88.1	85.7	69.0	89.3	88.1	86.9	88.1	88.1	88.1	88.1	88.1	88.1	88.1	86.9	86.9	85.7	85.7	85.7	86.9	86.9	88.1	86.9	**9**	Golden-headed lion tamarin AdV
**10**	28.7	8.9	11.6	13.0	11.6	8.9	23.6	36.0	14.4		90.5	90.5	88.1	88.1	90.5	90.5	83.3	79.8	83.3	83.3	82.1	84.5	83.3	86.9	89.3	83.3	92.9	89.3	75.0	88.1	88.1	90.5	88.1	88.1	88.1	88.1	88.1	88.1	88.1	88.1	88.1	86.9	89.3	89.3	88.1	88.1	89.3	91.7	**10**	Gray-bellied night monkey AdV
**11**	28.7	1.2	6.2	8.9	10.2	1.2	15.9	27.0	6.2	10.2		100.0	96.4	88.1	97.6	97.6	85.7	83.3	85.7	85.7	84.5	89.3	88.1	90.5	90.5	86.9	91.7	88.1	72.6	95.2	92.9	92.9	92.9	92.9	92.9	92.9	92.9	92.9	92.9	89.3	89.3	88.1	88.1	88.1	89.3	89.3	90.5	91.7	**11**	Marmoset AdV 1.1
**12**	28.7	1.2	6.2	8.9	10.2	1.2	15.9	27.0	6.2	10.2	0.0		96.4	88.1	97.6	97.6	85.7	83.3	85.7	85.7	84.5	89.3	88.1	90.5	90.5	86.9	91.7	88.1	72.6	95.2	92.9	92.9	92.9	92.9	92.9	92.9	92.9	92.9	92.9	89.3	89.3	88.1	88.1	88.1	89.3	89.3	90.5	91.7	**12**	Marmoset AdV 1.2
**13**	30.4	4.9	7.5	11.6	13.0	4.9	17.4	27.0	10.2	13.0	3.7	3.7		86.9	94.0	94.0	86.9	82.1	83.3	84.5	84.5	86.9	85.7	88.1	88.1	85.7	89.3	88.1	72.6	92.9	90.5	91.7	91.7	91.7	91.7	91.7	91.7	91.7	91.7	89.3	89.3	88.1	88.1	88.1	89.3	89.3	88.1	90.5	**13**	Red-bellied tamarin AdV
**14**	30.4	11.6	11.6	14.4	15.9	14.4	27.0	34.1	18.9	13.0	13.0	13.0	14.4		88.1	88.1	81.0	77.4	81.0	82.1	82.1	82.1	81.0	86.9	88.1	83.3	85.7	85.7	72.6	89.3	89.3	90.5	90.5	90.5	90.5	90.5	90.5	90.5	90.5	84.5	84.5	83.3	85.7	85.7	84.5	84.5	85.7	89.3	**14**	Red-faced spider monkey AdV
**15**	32.2	1.2	8.9	11.6	13.0	3.7	18.9	30.4	7.5	10.2	2.4	2.4	6.2	13.0		100.0	85.7	81.0	85.7	83.3	82.1	86.9	88.1	88.1	90.5	84.5	89.3	88.1	70.2	92.9	94.0	92.9	90.5	90.5	90.5	90.5	90.5	90.5	90.5	86.9	86.9	85.7	88.1	88.1	86.9	86.9	88.1	91.7	**15**	Red-handed tamarin AdV 2.1
**16**	32.2	1.2	8.9	11.6	13.0	3.7	18.9	30.4	7.5	10.2	2.4	2.4	6.2	13.0	0.0		85.7	81.0	85.7	83.3	82.1	86.9	88.1	88.1	90.5	84.5	89.3	88.1	70.2	92.9	94.0	92.9	90.5	90.5	90.5	90.5	90.5	90.5	90.5	86.9	86.9	85.7	88.1	88.1	86.9	86.9	88.1	91.7	**16**	Red-handed tamarin AdV 2.2
**17**	39.9	14.4	18.9	22.0	20.5	14.4	32.2	34.1	20.5	18.9	15.9	15.9	14.4	22.0	15.9	15.9		71.4	79.8	82.1	81.0	76.2	79.8	83.3	84.5	78.6	82.1	83.3	71.4	83.3	83.3	84.5	83.3	83.3	83.3	83.3	83.3	83.3	83.3	82.1	82.1	81.0	83.3	83.3	82.1	82.1	82.1	85.7	**17**	Red-handed tamarin AdV
**18**	17.4	20.5	15.9	20.5	22.0	20.5	3.7	17.4	27.0	23.6	18.9	18.9	20.5	27.0	22.0	22.0	36.0		79.8	75.0	77.4	82.1	79.8	78.6	78.6	79.8	82.1	78.6	78.6	83.3	81.0	82.1	84.5	84.5	84.5	84.5	84.5	84.5	84.5	79.8	79.8	79.8	78.6	78.6	79.8	79.8	81.0	83.3	**18**	Ring tailed lemur AdV
**19**	30.4	14.4	14.4	20.5	22.0	17.4	23.6	30.4	22.0	18.9	15.9	15.9	18.9	22.0	15.9	15.9	23.6	23.6		76.2	86.9	94.0	96.4	83.3	86.9	85.7	79.8	81.0	69.0	85.7	85.7	85.7	85.7	85.7	85.7	85.7	85.7	85.7	85.7	79.8	79.8	78.6	81.0	81.0	79.8	79.8	81.0	86.9	**19**	Simian AdV 1
**20**	34.1	17.4	15.9	17.4	17.4	15.9	30.4	39.9	17.4	18.9	15.9	15.9	17.4	20.5	18.9	18.9	20.5	30.4	28.7		77.4	79.8	76.2	82.1	82.1	78.6	85.7	82.1	71.4	84.5	82.1	83.3	84.5	84.5	84.5	84.5	84.5	84.5	84.5	83.3	83.3	82.1	82.1	82.1	83.3	83.3	84.5	83.3	**20**	Simian AdV 13
**21**	27.0	18.9	13.0	20.5	22.0	18.9	27.0	34.1	23.6	20.5	17.4	17.4	17.4	20.5	20.5	20.5	22.0	27.0	14.4	27.0		86.9	84.5	85.7	88.1	95.2	82.1	79.8	71.4	84.5	82.1	83.3	86.9	86.9	86.9	86.9	86.9	86.9	86.9	81.0	81.0	79.8	79.8	79.8	81.0	81.0	81.0	84.5	**21**	Simian AdV 16
**22**	28.7	13.0	10.2	15.9	17.4	13.0	20.5	30.4	17.4	17.4	11.6	11.6	14.4	20.5	14.4	14.4	28.7	20.5	6.2	23.6	14.4		95.2	85.7	84.5	88.1	83.3	82.1	69.0	89.3	86.9	86.9	89.3	89.3	89.3	89.3	89.3	89.3	89.3	83.3	83.3	82.1	82.1	82.1	83.3	83.3	84.5	88.1	**22**	Simian AdV 18
**23**	30.4	11.6	14.4	20.5	22.0	14.4	20.5	28.7	18.9	18.9	13.0	13.0	15.9	22.0	13.0	13.0	23.6	23.6	3.7	28.7	17.4	4.9		83.3	84.5	85.7	79.8	81.0	69.0	85.7	85.7	85.7	85.7	85.7	85.7	85.7	85.7	85.7	85.7	79.8	79.8	78.6	81.0	81.0	79.8	79.8	81.0	86.9	**23**	Simian AdV 19
**24**	32.2	11.6	10.2	11.6	10.2	8.9	25.3	32.2	14.4	14.4	10.2	10.2	13.0	14.4	13.0	13.0	18.9	25.3	18.9	20.5	15.9	15.9	18.9		89.3	88.1	89.3	88.1	73.8	91.7	89.3	89.3	90.5	90.5	90.5	90.5	90.5	90.5	90.5	89.3	89.3	88.1	88.1	88.1	89.3	89.3	90.5	90.5	**24**	Simian AdV 20
**25**	25.3	8.9	10.2	13.0	14.4	11.6	25.3	32.2	15.9	11.6	10.2	10.2	13.0	13.0	10.2	10.2	17.4	25.3	14.4	20.5	13.0	17.4	17.4	11.6		88.1	85.7	86.9	71.4	90.5	90.5	90.5	89.3	89.3	89.3	89.3	89.3	89.3	89.3	85.7	85.7	84.5	86.9	86.9	85.7	85.7	86.9	89.3	**25**	Simian AdV 3
**26**	27.0	15.9	11.6	17.4	18.9	15.9	27.0	34.1	20.5	18.9	14.4	14.4	15.9	18.9	17.4	17.4	25.3	23.6	15.9	25.3	4.9	13.0	15.9	13.0	13.0		83.3	82.1	73.8	86.9	84.5	84.5	88.1	88.1	88.1	88.1	88.1	88.1	88.1	83.3	83.3	82.1	82.1	82.1	83.3	83.3	83.3	85.7	**26**	Simian AdV 8
**27**	28.7	10.2	10.2	8.9	7.5	7.5	20.5	32.2	13.0	7.5	8.9	8.9	11.6	15.9	11.6	11.6	20.5	20.5	23.6	15.9	20.5	18.9	23.6	11.6	15.9	18.9		90.5	75.0	89.3	86.9	89.3	89.3	89.3	89.3	89.3	89.3	89.3	89.3	91.7	91.7	90.5	90.5	90.5	91.7	91.7	92.9	90.5	**27**	Simian AdV WIV19
**28**	36.0	11.6	13.0	3.7	2.4	11.6	25.3	32.2	15.9	11.6	13.0	13.0	13.0	15.9	13.0	13.0	18.9	25.3	22.0	20.5	23.6	20.5	22.0	13.0	14.4	20.5	10.2		71.4	90.5	90.5	90.5	89.3	89.3	89.3	89.3	89.3	89.3	89.3	98.8	98.8	97.6	100.0	100.0	98.8	98.8	97.6	91.7	**28**	Titi monkey AdV
**29**	27.0	36.0	34.1	34.1	32.2	32.2	28.7	30.4	39.9	30.4	34.1	34.1	34.1	34.1	37.9	37.9	36.0	25.3	39.9	36.0	36.0	39.9	39.9	32.2	36.0	32.2	30.4	36.0		75.0	72.6	73.8	72.6	72.6	72.6	72.6	72.6	72.6	72.6	72.6	72.6	72.6	71.4	71.4	72.6	72.6	73.8	72.6	**29**	Tree shrew AdV-1
**30**	30.4	6.2	6.2	6.2	7.5	6.2	18.9	27.0	11.6	13.0	4.9	4.9	7.5	11.6	7.5	7.5	18.9	18.9	15.9	17.4	17.4	11.6	15.9	8.9	10.2	14.4	11.6	10.2	30.4		97.6	97.6	95.2	95.2	95.2	95.2	95.2	95.2	95.2	91.7	91.7	90.5	90.5	90.5	91.7	91.7	92.9	94.0	**30**	Tufted capuchin AdV 1
**31**	34.1	6.2	8.9	8.9	10.2	8.9	22.0	30.4	13.0	13.0	7.5	7.5	10.2	11.6	6.2	6.2	18.9	22.0	15.9	20.5	20.5	14.4	15.9	11.6	10.2	17.4	14.4	10.2	34.1	2.4		97.6	92.9	92.9	92.9	92.9	92.9	92.9	92.9	89.3	89.3	88.1	90.5	90.5	89.3	89.3	90.5	94.0	**31**	Tufted capuchin AdV 2
**32**	32.2	6.2	7.5	8.9	10.2	8.9	20.5	28.7	14.4	10.2	7.5	7.5	8.9	10.2	7.5	7.5	17.4	20.5	15.9	18.9	18.9	14.4	15.9	11.6	10.2	17.4	11.6	10.2	32.2	2.4	2.4		94.0	94.0	94.0	94.0	94.0	94.0	94.0	89.3	89.3	88.1	90.5	90.5	89.3	89.3	90.5	96.4	**32**	Tufted capuchin AdV 3
**33**	27.0	8.9	1.2	7.5	8.9	8.9	17.4	25.3	13.0	13.0	7.5	7.5	8.9	10.2	10.2	10.2	18.9	17.4	15.9	17.4	14.4	11.6	15.9	10.2	11.6	13.0	11.6	11.6	34.1	4.9	7.5	6.2		100.0	100.0	100.0	100.0	100.0	100.0	90.5	90.5	89.3	89.3	89.3	90.5	90.5	91.7	95.2	**33**	Squirrel monkey AdV 1.0
**34**	27.0	8.9	1.2	7.5	8.9	8.9	17.4	25.3	13.0	13.0	7.5	7.5	8.9	10.2	10.2	10.2	18.9	17.4	15.9	17.4	14.4	11.6	15.9	10.2	11.6	13.0	11.6	11.6	34.1	4.9	7.5	6.2	0.0		100.0	100.0	100.0	100.0	100.0	90.5	90.5	89.3	89.3	89.3	90.5	90.5	91.7	95.2	**34**	Squirrel monkey AdV 1.1
**35**	27.0	8.9	1.2	7.5	8.9	8.9	17.4	25.3	13.0	13.0	7.5	7.5	8.9	10.2	10.2	10.2	18.9	17.4	15.9	17.4	14.4	11.6	15.9	10.2	11.6	13.0	11.6	11.6	34.1	4.9	7.5	6.2	0.0	0.0		100.0	100.0	100.0	100.0	90.5	90.5	89.3	89.3	89.3	90.5	90.5	91.7	95.2	**35**	Squirrel monkey AdV 1.2
**36**	27.0	8.9	1.2	7.5	8.9	8.9	17.4	25.3	13.0	13.0	7.5	7.5	8.9	10.2	10.2	10.2	18.9	17.4	15.9	17.4	14.4	11.6	15.9	10.2	11.6	13.0	11.6	11.6	34.1	4.9	7.5	6.2	0.0	0.0	0.0		100.0	100.0	100.0	90.5	90.5	89.3	89.3	89.3	90.5	90.5	91.7	95.2	**36**	Squirrel monkey AdV 1.3
**37**	27.0	8.9	1.2	7.5	8.9	8.9	17.4	25.3	13.0	13.0	7.5	7.5	8.9	10.2	10.2	10.2	18.9	17.4	15.9	17.4	14.4	11.6	15.9	10.2	11.6	13.0	11.6	11.6	34.1	4.9	7.5	6.2	0.0	0.0	0.0	0.0		100.0	100.0	90.5	90.5	89.3	89.3	89.3	90.5	90.5	91.7	95.2	**37**	Squirrel monkey AdV 1.4
**38**	27.0	8.9	1.2	7.5	8.9	8.9	17.4	25.3	13.0	13.0	7.5	7.5	8.9	10.2	10.2	10.2	18.9	17.4	15.9	17.4	14.4	11.6	15.9	10.2	11.6	13.0	11.6	11.6	34.1	4.9	7.5	6.2	0.0	0.0	0.0	0.0	0.0		100.0	90.5	90.5	89.3	89.3	89.3	90.5	90.5	91.7	95.2	**38**	Squirrel monkey AdV 1.9
**39**	27.0	8.9	1.2	7.5	8.9	8.9	17.4	25.3	13.0	13.0	7.5	7.5	8.9	10.2	10.2	10.2	18.9	17.4	15.9	17.4	14.4	11.6	15.9	10.2	11.6	13.0	11.6	11.6	34.1	4.9	7.5	6.2	0.0	0.0	0.0	0.0	0.0	0.0		90.5	90.5	89.3	89.3	89.3	90.5	90.5	91.7	95.2	**39**	Squirrel monkey AdV 1.11
**40**	34.1	13.0	11.6	2.4	1.2	10.2	23.6	30.4	14.4	13.0	11.6	11.6	11.6	17.4	14.4	14.4	20.5	23.6	23.6	18.9	22.0	18.9	23.6	11.6	15.9	18.9	8.9	1.2	34.1	8.9	11.6	11.6	10.2	10.2	10.2	10.2	10.2	10.2	10.2		100.0	98.8	98.8	98.8	100.0	100.0	98.8	90.5	**40**	Squirrel monkey AdV 2.1
**41**	34.1	13.0	11.6	2.4	1.2	10.2	23.6	30.4	14.4	13.0	11.6	11.6	11.6	17.4	14.4	14.4	20.5	23.6	23.6	18.9	22.0	18.9	23.6	11.6	15.9	18.9	8.9	1.2	34.1	8.9	11.6	11.6	10.2	10.2	10.2	10.2	10.2	10.2	10.2	0.0		98.8	98.8	98.8	100.0	100.0	98.8	90.5	**41**	Squirrel monkey AdV 2.3
**42**	34.1	14.4	13.0	3.7	2.4	11.6	23.6	30.4	15.9	14.4	13.0	13.0	13.0	18.9	15.9	15.9	22.0	23.6	25.3	20.5	23.6	20.5	25.3	13.0	17.4	20.5	10.2	2.4	34.1	10.2	13.0	13.0	11.6	11.6	11.6	11.6	11.6	11.6	11.6	1.2	1.2		97.6	97.6	98.8	98.8	97.6	89.3	**42**	Squirrel monkey AdV 2.5
**43**	36.0	11.6	13.0	3.7	2.4	11.6	25.3	32.2	15.9	11.6	13.0	13.0	13.0	15.9	13.0	13.0	18.9	25.3	22.0	20.5	23.6	20.5	22.0	13.0	14.4	20.5	10.2	0.0	36.0	10.2	10.2	10.2	11.6	11.6	11.6	11.6	11.6	11.6	11.6	1.2	1.2	2.4		100.0	98.8	98.8	97.6	91.7	**43**	Squirrel monkey AdV 2.7
**44**	36.0	11.6	13.0	3.7	2.4	11.6	25.3	32.2	15.9	11.6	13.0	13.0	13.0	15.9	13.0	13.0	18.9	25.3	22.0	20.5	23.6	20.5	22.0	13.0	14.4	20.5	10.2	0.0	36.0	10.2	10.2	10.2	11.6	11.6	11.6	11.6	11.6	11.6	11.6	1.2	1.2	2.4	0.0		98.8	98.8	97.6	91.7	**44**	Squirrel monkey AdV 2.12
**45**	34.1	13.0	11.6	2.4	1.2	10.2	23.6	30.4	14.4	13.0	11.6	11.6	11.6	17.4	14.4	14.4	20.5	23.6	23.6	18.9	22.0	18.9	23.6	11.6	15.9	18.9	8.9	1.2	34.1	8.9	11.6	11.6	10.2	10.2	10.2	10.2	10.2	10.2	10.2	0.0	0.0	1.2	1.2	1.2		100.0	98.8	90.5	**45**	Squirrel monkey AdV 2.14
**46**	34.1	13.0	11.6	2.4	1.2	10.2	23.6	30.4	14.4	13.0	11.6	11.6	11.6	17.4	14.4	14.4	20.5	23.6	23.6	18.9	22.0	18.9	23.6	11.6	15.9	18.9	8.9	1.2	34.1	8.9	11.6	11.6	10.2	10.2	10.2	10.2	10.2	10.2	10.2	0.0	0.0	1.2	1.2	1.2	0.0		98.8	90.5	**46**	Squirrel monkey AdV 2.15
**47**	32.2	11.6	10.2	1.2	0.0	8.9	22.0	30.4	13.0	11.6	10.2	10.2	13.0	15.9	13.0	13.0	20.5	22.0	22.0	17.4	22.0	17.4	22.0	10.2	14.4	18.9	7.5	2.4	32.2	7.5	10.2	10.2	8.9	8.9	8.9	8.9	8.9	8.9	8.9	1.2	1.2	2.4	2.4	2.4	1.2	1.2		91.7	**47**	Squirrel monkey AdV 2.16
**48**	30.4	7.5	6.2	10.2	8.9	7.5	18.9	27.0	14.4	8.9	8.9	8.9	10.2	11.6	8.9	8.9	15.9	18.9	14.4	18.9	17.4	13.0	14.4	10.2	11.6	15.9	10.2	8.9	34.1	6.2	6.2	3.7	4.9	4.9	4.9	4.9	4.9	4.9	4.9	10.2	10.2	11.6	8.9	8.9	10.2	10.2	8.9		**48**	Squirrel monkey AdV 3
	**1**	**2**	**3**	**4**	**5**	**6**	**7**	**8**	**9**	**10**	**11**	**12**	**13**	**14**	**15**	**16**	**17**	**18**	**19**	**20**	**21**	**22**	**23**	**24**	**25**	**26**	**27**	**28**	**29**	**30**	**31**	**32**	**33**	**34**	**35**	**36**	**37**	**38**	**39**	**40**	**41**	**42**	**43**	**44**	**45**	**46**	**47**	**48**		

Figure S2. Amino acid pairwise identity comparison of squirrel monkey adenoviruses to adenoviruses of NWMs, other primates and the tree shrew, based on partial sequences of the IVa2 gene

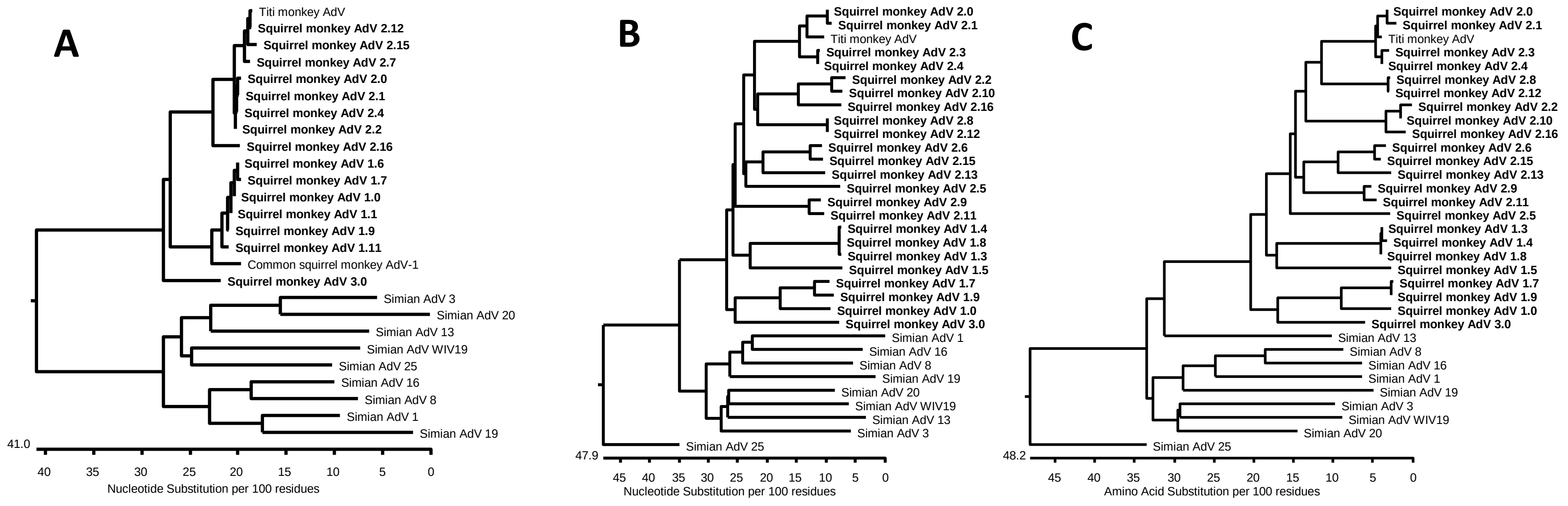

Figure S3. Phylogeny of adenoviruses detected in this study. Comparison of squirrel monkey adenoviruses to adenoviruses other primates. **A.** Comparison based on 288 nt the 3' end of the DNA polymerase. **B.** Comparison based on 694 - 792 nt of the hypervariable region of the hexon gene. **C.** Comparison based on 231 – 247 aa of the hypervariable region of the hexon gene. Adenoviruses detected in this study are bolded.
